# Characterizing the Contribution of Strain Specificity to the Microbiota Structure and Metabolites of *Muqu* and Fresh High-Temperature *Daqu*

**DOI:** 10.3390/foods13193098

**Published:** 2024-09-27

**Authors:** Yi Zhang, Zhu Zhang, Jun Huang, Rongqing Zhou, Qiuxiang Tang, Yao Jin

**Affiliations:** College of Biomass Science and Engineering, Sichuan University, Chengdu 610065, China; yizhangscu@163.com (Y.Z.); 2021223085074@stu.scu.edu.cn (Z.Z.); huangjun@scu.edu.cn (J.H.); zhourqing@scu.edu.cn (R.Z.); tangqiuxiang@scu.edu.cn (Q.T.)

**Keywords:** high-temperature *Daqu*, microbial community, physicochemical properties, metabolites profiles, multiphase detection technique

## Abstract

In this study, the differences in physicochemical properties, microbial community structure, and metabolic characteristics between various fortified *Muqu* and their corresponding high-temperature *Daqu* (HTD) were investigated using multiphase detection methods. The results demonstrated that the physicochemical properties, community structure, dominant bacterial composition, and metabolic components varied significantly among the different types of fortified HTD. The differences between HTDs became more pronounced when fortified HTD was used as *Muqu*. Compared to HTD, *Muqu* exhibited a more complex volatile profile, while HTD contained higher levels of characteristic non-volatile components. The cultivable bacteria count in *Muqu* was significantly higher than that in HTD, while the cultivable fungi count was slightly lower than that in HTD. The fungal profiles in HTD were primarily associated with starch hydrolysis and ethanol synthesis, while bacterial activity was more prominent in *Muqu*. Additionally, pyrazine synthesis was mainly attributed to fungi in *Muqu* and bacteria in HTD. Source Tracker analysis indicated that 8.11% of the bacteria and 26.76% of the fungi originated from *Muqu*. This study provides a theoretical foundation for the controlled production of HTD, contributing to improvements in its quality and consistency.

## 1. Introduction

*Daqu* is irreplaceable in traditional Chinese conventional fermented foods, serving as a unique fermentation starter and raw material. The special texture structure, flavor constituents, and their precursors are attributed to traditional craftsmanship. High-temperature *Daqu* (HTD), also known as *Maotai Daqu* or *Jiangxiang Daqu*, is one of the three basic traditional *Daqu* used for *Baijiu* fermentation. The quality of HTD, which is used in quantities slightly exceeding those of sorghum, significantly contributes to the characteristic flavor and yield of fresh sauce soy *Baijiu* [1,2]. Unlike *Nongxiang Daqu*, HTD processing involves the addition of *Muqu* (high-quality *Daqu* from the previous year), along with variations in process parameters [3]. Previous studies suggest that it can improve *Daqu* quality by colonizing certain functional strains preferentially. However, the contribution of the microbiota in *Muqu* to microbiota in different types of HTD remains unclear [4]. Improving *Daqu* quality remains challenging as it is traditionally controlled by artisans based on their experiences. Since *Daqu* is manufactured in an open environment, its quality and properties are influenced by both biotic and abiotic factors, resulting in significant temporal and spatial variability [5,6].

Emerging omics and bioinformatics provide powerful tools to understand the role of microbiota in *Daqu*. For example, the dominant microbiota in HTD include *Thermoactinomycetaceae* and *Bacillaceae*, with *Bacillus* as a keystone genus [7]. *Monascus* is also a dominant fungus, accounting for more than 50% of the relative abundance (RA) in the mid and later phases of HTD processing. Specifically, *Monascus* accounts for 21.1% and 30.3% of Yellow Qu and White Qu, respectively [8]. The presence of *Monascus* is positively correlated with the activities of α-amylase, glucoamylase, cellulase, and protease [9]. Additionally, isolates of *Monascus purpureus* YJX-8 synthesize short-chain fatty acid esters in the aqueous phase, imparting a unique flavor to fresh *Baijiu*, with the enzyme LIP05 belonging to the α/β hydrolase family [10]. Among *Nongxiang Daqu* and *Qingxiang Daqu* (QXD), both share the same dominant genera, such as *Bacillus* and *Lactobacillus*. However, they also have unique genera, such as *Asperigillus* and *Saccharomycopsis* in the former and *Saccharomyces* and *Rhizopus* in the latter [11,12].

These findings have laid an important foundation for the development of new technologies to improve *Daqu* quality, particularly through functional strain and microbiota. For instance, inoculating *Bacillus* spp. significantly enhanced the content of unique components such as ligustrazine and phenylethanol, thereby improving *Daqu* quality [13,14]. The *Bacillus licheniformis*, isolated from HTD, was inoculated into HTD and QXD as a starter, which endowed them with a unique flavor [14,15]. Moreover, the contents of aromatics, phenols, and pyrazines in fresh *Baijiu* were increased by 2.4, 0.5, and 3.9 times, respectively, in fortified HTD. Meanwhile, amylase activity and the content of pyrazines and aromatic compounds were notably increased in fortified *Daqu* [15,16]. However, few reports exist on the effect of fortified HTD with *Monascus* used in *Muqu* on the microbiota and their metabolites in matured HTD [17].

In this study, the effects of fortified HTD with *Monascus*-based *Muqu* on physicochemical properties, microbial community structure and diversity, as well as metabolites, were investigated by a multiphase detection approach, including conventional analysis methods, chromatography-mass spectrometry, as well as the Illumina MiSeq platform. Meanwhile, the contribution of the dominant microbiota in *Muqu* to mature HTD was explored with Source Tracker software 1.0.1. Relationships among physicochemical properties, metabolic components, and dominant microorganisms were analyzed by Spearman’s and Mantel’s tests. This provided a theoretical basis for the controllable production of HTD.

## 2. Materials and Methods

### 2.1. Preparation and Sampling of Fortified High-Temperature Daqu

#### 2.1.1. Microorganism

Three isolates of *Monascus floridanus* were obtained from high-temperature *Daqu*. These isolates were identified based on their ITS sequences and preserved in our laboratory. They were named H14, H26, and H30, respectively.

#### 2.1.2. Seed Preparation

The seed preparation followed these steps: These isolated strains were inoculated onto malt extract agar medium (with a sugar concentration of 8°Brix) and cultivated at 30 °C for five days, followed by washing with a sterile 2% acetic acid solution. The spores of activated isolates (5.45 × 10^5^ spores/g) were inoculated into wheat flour medium, which had been autoclaved for 20 min at 121 °C. This process was performed using 500 g of wheat flour in a 5 L triangular flask. The cultures were incubated at 30 ± 1 °C for 3–5 d, then dried until the moisture content was reduced to below 13% at a temperature range of 40–45 °C.

#### 2.1.3. *Muqu* Production

The *Muqu* was produced using the traditional method described by Zhu et al. [2]. In brief, crushed wheat was thoroughly mixed with water, and 3% (*w/w*, dried weight) of the respective seed powder. The mixture was then shaped into *Qupei* blocks (with a moisture content of 37%) and stacked into piles with four layers. During the fermentation process, the piles were flipped on day 7 and day 14. Fermentation was completed on day 40, after which HTD was transferred to a storage room and stored for 6 months at ambient temperature. The matured HTD fortified with isolate H14, H26, and H30 were used as *Muqu* for the subsequent round of HTD, and each was labeled as H14-M, H26-M, and H30-M, respectively.

#### 2.1.4. High-Temperature *Daqu* Production and Sampling

The HTD production process followed the traditional method described by Zhu et al. [2] with the *Muqu* replaced by 6% of H14-M, H26-M, and H30-M, respectively. The resulting HTD products were labeled as H14-ZF, H26-ZF, and H30-ZF, respectively. Three pieces of HTD from each group were randomly sampled, crushed, mixed evenly, and stacked conically. A 500 g sample was taken using a five-point sampling approach. The samples were divided into 100 g and 400 g portions and stored at −20 °C and −80 °C, respectively, until further analysis.

### 2.2. Determination of Physiochemical Properties

The moisture, saccharification ability, liquefaction ability, fermentation ability, esterification ability, acidity, and ammoniacal nitrogen were determined according to the general methods of analysis for *Daqu* as specified in QB/T 4257-2011 [18]. Specifically, the moisture content was determined by drying the *Daqu* sample at 105 °C until a constant weight was achieved, with the calculation based on the mass difference before and after drying. Saccharification ability was assessed by measuring the milligrams of glucose produced from the conversion of soluble starch by 1.0 g of absolutely dry sample per hour under conditions of 35 °C and pH 4.6. The liquefaction ability was measured by determining the grams of starch liquefied by 1.0 g of absolutely dry sample per hour under the same temperature and pH conditions. The fermentation ability was evaluated by fermenting 0.5 g of absolutely dry sample at 30 °C for 72 h and measuring the volume of CO_2_ produced during the fermentation process. The esterification ability was determined by measuring the amount of ethyl hexanoate produced by catalyzing ethanol and hexanoic acid in 50 g of absolutely dry *Daqu* over a period of 7 d at 35 °C. Acidity was measured using a titration method with acid-base neutralization, where the endpoint was indicated by a change in pH, to determine the acidity of the *Daqu*. The ammonium nitrogen content was determined based on the amphoteric nature of amino acids. This involved reacting the sample with a standard solution of sodium hydroxide, which neutralized the basicity of the amino groups, leaving the carboxyl groups acidic. The titration endpoint was controlled with a pH indicator, and the ammonium nitrogen content was calculated based on the volume of sodium hydroxide standard solution consumed.

### 2.3. Detection of Volatile Metabolic Components

A sample of 1.000 g of the *Daqu* was accurately weighed and placed in a 25 mL headspace vial. To this, 10 µL of the internal standard substance (Caprylic acid methyl ester: 0.0079 g/100 mL) was added. The vial was then placed on a magnetic stirrer at a constant temperature of 60 ± 1 °C and allowed to equilibrate for 15 min. A solid-phase microextraction (SPME) fiber (50/30 µm, DVB/CAR/PDMS) was inserted into the headspace vial and exposed for extraction for 45 min. After extraction, the fiber was inserted into the gas chromatograph (GC) injection port for desorption for 5 min.

GC conditions: The temperature of the injection port was set to 250 °C, and no split mode was used. Temperature program began at 40 °C, held for 5 min, then increased at 5 °C/min to 220 °C and held for 5 min. High-purity helium (>99.999%) was used as the carrier gas at a constant flow rate of 1 mL/min. MS conditions: The interface temperature was set to 250 °C, and Electron Ionization (EI) mode was used with an electron energy of 70 eV. The ion source temperature was 200 °C with a mass spectrometer set to scan over a range of 30–400 amu at a scan rate of 1 scan/s. Volatile compounds were identified by comparing their mass spectrum with data in the NIST2017 library database (Finnigan Co., San Jose, CA, USA) based on a similarity index > 800.

### 2.4. Detection of Non-Volatile Metabolic Components

Non-volatile metabolites were detected using ultra-performance liquid chromatography coupled with quadrupole time-of-flight mass spectrometry (UPLC-Q/TOF-MS). The system included a 1290 Infinity II equipped with a Zorbax Eclipse Plus C18 column (2.1 mm × 50 mm, 1.9 μm), Q/TOF-MS (G6546A), MassHunter Profinder (v10.0.2), MassHunter Qualitative Analysis (v10.0), and PCDL Manager B.08.00.

#### 2.4.1. Sample Preparation

A 5.00 g sample was mixed with 20 mL of 1% (*w*/*v*) CaCl_2_, and the mixture was allowed to stand overnight at 4 °C. The mixture was then subjected to ultrasonication in an ice bath for 30 min. After centrifugation at 4 °C and 8000 rpm for 20 min, the supernatant was collected, and 10 μL of the internal standard (2-chloro-L-phenylalanine, 104 μg/L) was added. The residue was vacuum freeze-dried, dissolved in 10 mL of an 80% methanol solution, vortexed, and filtered through a 0.22 μm nylon filter membrane. The filtrate was transferred to an injection vial.

#### 2.4.2. UPLC Conditions

Automatic injection of 2 µL of supernatant was performed. Gradient elution was carried out with 0.1% formic acid aqueous solution (A) and acetonitrile solution (B) at a flow rate of 0.3 mL/min using the following program: 0–5 min, 98% A; 5–13 min, 98% to 90% A; 13–23 min, 90% to 5% A; 23–26 min, 5% A; 26–26.1 min, 5% to 98% A; and 26.1–30 min, 5% to 98%.

#### 2.4.3. MS Conditions

A triple quadrupole time-of-flight mass spectrometer with a Dual AJS ESI ion source was used. The capillary voltages were set to 4.0 kV for positive mode and 3.5 kV for negative mode. The mass scan range was m/z 100–1000, with a full scan at a resolution of 60,000. Auto MS/MS experiments were conducted using collision-induced dissociation (CID) scanning with normalized collision energies of 20 eV and 40 eV. Additional MS conditions included a curtain gas temperature of 300 °C, a gas flow rate of 10 L/min, a nebulizer pressure of 60 psi, a sheath gas temperature of 350 °C, and a sheath gas flow rate of 11 L/min.

Raw data generated by MassHunter Profinder (v10.0.2) was used for peak alignment, peak picking, peak matching, and semi-quantitative analysis of each metabolite. The main parameters for peak alignment included a retention time and mass tolerance of 0.30 min and 20 ppm, respectively. Retention time and mass tolerance for peak matching were 0.15 min and 10 ppm, respectively, with peaks having an absolute height of ≥ 20,000 and a core of ≥70. Subsequently, MassHunter Qualitative Analysis (v10.0) software was used for automatic MS/MS library searches and online database searches to obtain accurate qualitative results.

### 2.5. Enumeration of Cultivable Microorganisms in Daqu

A 5.00 g *Daqu* sample was placed in a triangular flask containing 100 mL of sterile normal saline and a glass bead was thoroughly mixed. These suspensions were serially diluted, plated onto beef extract peptone agar medium and Bengal red medium, then incubated at 37 °C for 36–48 h for the total bacteria count, and at 30 °C for 48–72 h for the fungal count. For mesophilic bacteria, the suspension was treated for 10 min at 80 °C in a water bath before incubation [19,20]. The sample was then spread on beef extract peptone agar medium, incubated at 37 ± 1 °C for 36–48 h, and the colonies were counted.

### 2.6. Analysis of Daqu Microbial Community Structure

Total DNA was extracted from *Daqu* using the Fast DNA SPIN extraction kit (MP Biomedicals, Santa Ana, CA, USA) following the manufacturer’s instructions. DNA concentration and purity were assessed using a Nanodrop DN-1000 spectrophotometer and 1% agarose gel electrophoresis. PCR amplification and sequencing: The V3-V4 region of the bacterial 16S rRNA gene and the ITS1 and ITS5 regions of the fungal genome were amplified using primers 338F/806R and ITS5F/ITS1R, respectively. The PCR system (25 µL) consisted of 5 µL each of 5 × reaction buffer and 5 × GC buffer, 0.25 µL of High-Fidelity DNA polymerase (5 U/µL), 2 µL of dNTPs (2.5 mmol/L), 1 µL each of forward and reverse primers (10 µmol/L), 2 µL of DNA template, and 8.57 µL of ddH₂O. PCR conditions: For bacteria, the PCR conditions were as follows: preheating at 98 °C for 2 min, followed by 25 cycles of denaturation at 98 °C for 15 s, annealing at 55 °C for 30 s, with a final extension at 72 °C for 30 s. For fungi, the PCR conditions were as follows: preheating at 95 °C for 3 min, followed by 32 cycles of denaturation at 95 °C for 45 s, annealing at 61 °C for 30 s, and extension at 72 °C for 10 min.

After PCR amplification, the products were purified using agarose gel electrophoresis and gel extraction. The purified fragments are then further purified using the Agencourt AMPure Beads nucleic acid purification kit and sequenced by Shanghai Personal Biotechnology Co., Ltd. (Shanghai, China) based on the 2 × 300 Illumina MiSeq platform.

### 2.7. Data Analysis

Data analysis was performed using the Tutool analysis platform (https://www.cloudtutu.com (accessed on 5 June 2024)) for principal coordinate analysis (PCoA), principal component analysis (PCA), heatmap clustering analysis, linear discriminant analysis effect size (LEfSe), mantel test, and redundancy analysis correlation analysis. Differential analyses of metabolite components were conducted using partial least squares discriminant analysis (PLS-DA) in SIMCA 14.1. Statistical significance was assessed using Duncan’s test within one-way ANOVA in SPSS 25.0 software (*p* < 0.05, n = 3). Source tracking analysis was performed using the Source Tracker to evaluate the microbial origins of the HTD.

## 3. Results and Discussion

### 3.1. Differences in Physicochemical Properties between Muqu and HTD

As shown in Table 1, significant differences in moisture content were observed among the HTD samples, with H30-ZF exhibiting the highest moisture content at 18.11 g/100 g. In contrast, only slight variations in moisture content were noted among the *Muqu* samples. The enzyme activities of *Muqu* and HTD did not exhibit positive correlations. HTD samples generally displayed reduced acidity and increased ammoniacal nitrogen levels, except for H14-ZF, which showed marginal fluctuations. In the case of H30-M, which corresponds to H30-ZF, a slight reduction in starch hydrolysis capacity was observed. However, the fermentation ability of HTD was notably enhanced compared to their respective *Muqu* counterparts, with H14-ZF showing a significant increase from 0.11 g/0.5 g·72 h to 0.23 g/0.5 g·72 h. On the other hand, esterification ability significantly declined in H14-ZF, with H26-ZF and H30-ZF showing slight decreases and increases, respectively.

### 3.2. Differences in Volatile Metabolites

**Figure 1:**
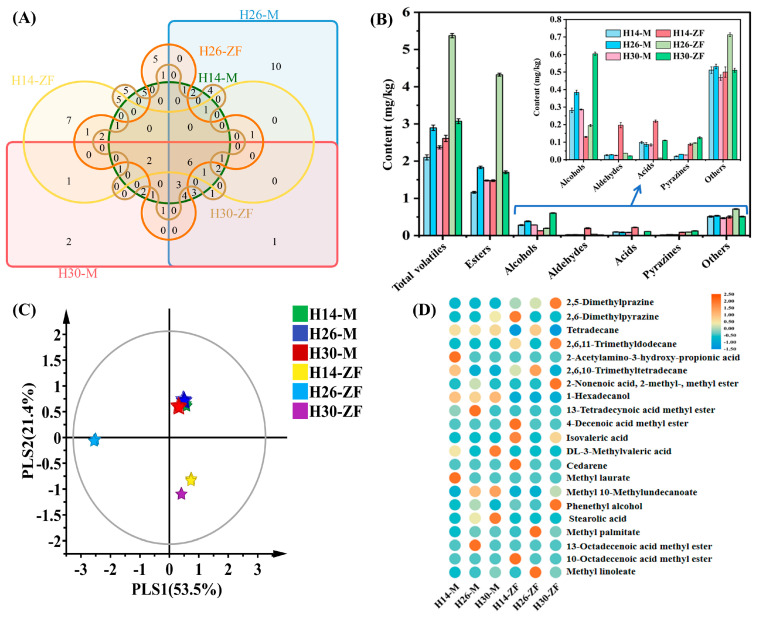
(**A**) Venn diagrams of volatile fractions of different *Daqu*; (**B**) volatile fraction content; (**C**) PLS-DA of volatiles; (**D**) heatmap displaying characteristic volatile metabolites.

Figure 1A,B illustrates that a total of 83 volatile metabolic components were identified across the samples, categorized into esters (**31**), alcohols (**9**), aldehydes (**7**), acids (**11**), pyrazines (**6**), and other compounds (**19**). Common components included phenethyl alcohol, undecane, dodecane, methyl palmitate, methyl linoleate, and methyl phenylacetate. The proportion of dominant esters ranged from 55.35% to 80.46%, with methyl palmitate and methyl linoleate accounting for 27.11% to 42.08% and 13.39% to 25.75% of the total volatile components, respectively. H14-M, H26-M, and H30-M shared 15, 16, and 14 components, respectively, with their corresponding HTD samples. H14-M, H26-M, and H30-M contained 36, 41, and 32 components, respectively, while corresponding HTD samples contained 32, 30, and 32 components, with their contents having increased by 24.31%, 85.62%, and 29.66%, respectively. The total content of volatile components in H26-ZF increased from 2.89 mg/kg to 5.37 mg/kg, with the esters increasing from 1.83 mg/kg to 4.32 mg/kg. Notably, the content of alcohol components in H14-ZF and H26-ZF significantly decreased, while the aldehyde and acid components in the former increased by 6.49 and 1.24 times, respectively, and the alcohol in H30-ZF increased by 1.11 times. Among the six main characteristic components identified in the *Baijiu*, including 2,5-dimethylpyrazine, 2,6-dimethylpyrazine, 2,3-dimethylpyrazine, 2-ethyl-6-methylpyrazine, 2,3,5,6-tetramethylpyrazine and 2,5-dimethyl-3-butylpyrazine, the levels in the corresponding HTD increased by 3.46, 2.09, and 3.64 times, respectively. Despite these differences, important components present in *Muqu* were found in HTD, though their number and concentration varied significantly.

Figure 1C shows the PLS-DA differential analysis of volatile components. The model quality, as evaluated by R2X, R2Y, and Q2 was close to 1, indicating that the model was robust and reliable. *Muqu* samples were located in the first quadrant, exhibiting relatively close proximity to each other, while the corresponding HTD samples were located in the third and fourth quadrants, indicating significant alterations in the volatile component profiles between *Muqu* and HTD. A total of 21 differential volatile metabolites were identified based on the criterion of VIP (Variable Importance in the Projection) > 1.0 (Figure 1D). These included 2,5-dimethylpyrazine, 2,6-dimethylpyrazine, 1-hexadecanol, phenethyl alcohol, isovaleric acid, 3-methylvaleric acid, stearolic acid, 2-acetamido-3-hydroxypropionic acid, cedarene, three alkanes, and nine fatty acid methyl esters. Phenethyl alcohol, methyl palmitate, and isovaleric acid were also significant components in *Daqu* and *Baijiu* [21,22]. Additionally, 1-Hexadecanol is a precursor of palmitic acid and ethyl palmitate [23], while cedarene is an important flavor terpenoid compound in the *Baijiu* [24].

### 3.3. Variation of Non-Volatile Components

**Figure 2:**
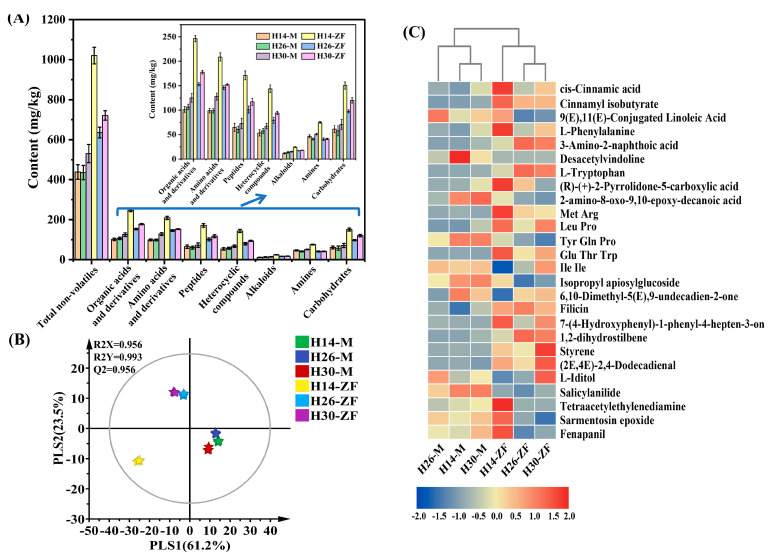
(**A**) The content of non-volatiles in different *Daqu*; (**B**) PLS-DA of non-volatiles; (**C**) heatmap displaying characteristic non-volatile components.

A total of 8233 and 2457 ion characteristic peaks were detected under the ESI^+^ and ESI^−^ modes of LC-Q/TOF-MS, respectively. Based on the MS/MS search, 568 reliable non-volatile components were identified, including 310 in ESI^+^ and 258 in ESI^−^. These substances were categorized into seven groups: organic acids and their derivatives (**125**), amino acids and their derivatives (**74**), peptides (**140**), heterocyclic compounds (**94**), alkaloids (**22**), amines (**36**), and carbohydrates (**77**). Similar to the trend observed in volatile components, the content of non-volatile components in HTD was significantly higher than that in the corresponding *Muqu* (Figure 2A). Notably, the total contents of H30-M and H14-ZF were 530.50 mg/kg and 1020.58 mg/kg, respectively, the highest among *Muqu* and HTD samples. The contents of non-volatile components in HTD exceeded those in *Muqu* across most categories, except for amines. Organic acids and their derivatives, as well as amino acids and their derivatives, accounted for more than 45% of the total non-volatile content, while peptides, heterocyclic compounds, and carbohydrates made up over 39%, with amines and alkaloids contributing slightly over 8%. These results confirmed that the non-volatile metabolic component profiles in HTD differ significantly from those in their respective *Muqu*.

The PLS-DA analysis of the characteristic non-volatile metabolic components yielded results that were similar to those for volatiles (Figure 2B). The structural differences in the characteristic metabolites of HTD were significant, with samples clustering in the second and third quadrants, and being relatively distant from each other. Based on the criterion of VIP > 2.0, 26 characteristic components were identified, including organic acids and their derivatives, amino acids, and their derivatives. Most characteristic components of HTD were present at higher levels than that in the corresponding *Muqu* samples. The cluster analysis results indicated that HTD and *Muqu* formed a distinct cluster (Figure 2C), with higher similarities observed between H30-ZF and H26-ZF, as well as between H30-M and H14-M.

### 3.4. Differences in Microbial Community Structure

Differences in the total bacterial count, mesophilic bacteria, and fungi among samples, as determined by the plate count method (Table 2). The total bacterial count in HTD was lower compared to *Muqu*, while the number of fungi increased. Moreover, the composition and proportion of mesophilic bacteria were altered, shifting from 3.20–9.74% to 55.08–78.26%. This shift may be driven by process parameters that likely influenced the evolution of the community structure.

**Figure 3:**
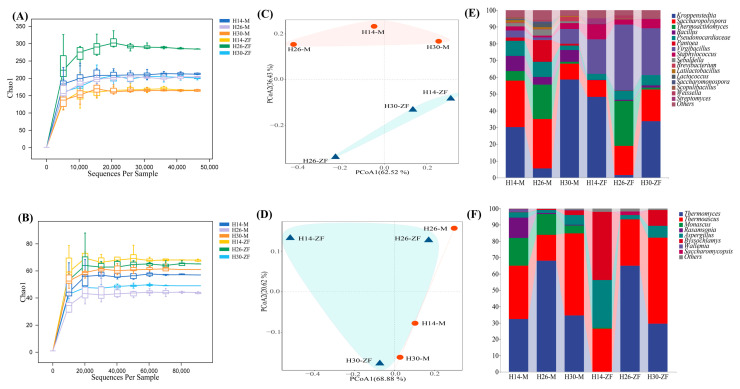
Rarefaction curves of (**A**) bacterial communities and (**B**) fungal communities; differences in β−diversity of (**C**) bacterial communities and (**D**) fungal communities; composition of microbial communities at genus level of (**E**) bacterial communities and (**F**) fungal communities.

The effective sequencing range for bacteria and fungi obtained from six types of *Daqu* was 91,574 to 115,317 and 123,261 to 138,732, respectively. The average values and proportions of high-quality sequences were 73,310 and 126,616, with 95.65% and 97.90%, respectively. The rarefaction curves for the six types of *Daqu* approached a plateau, indicating that the sequencing volume for each sample was reasonable and valid (Table 3, Figure 3A,B). The sequencing depth adequately represented the microbial composition of the samples.

A total of 147 bacterial genera and 69 fungal genera, each with RA > 1%, were identified. The distribution of genera in *Muqu* and HTD is presented in Figure 3E,F. *Muqu* samples contained 11, 12, and 9 bacterial genera, with 9 genera being common across the samples, aligning with previous reports on dominant bacteria. *Lactobacillus* was identified in H14-M and H26-M, with *Scoulibacillus*, *Lactococcus,* and *Saccharomonospora* being dominated in H14-M and H26-M, respectively, while *Brevibacterium* was dominant in H30-M. In the corresponding HTD, five to seven dominant bacterial genera were identified. In H14-ZF, *Bacillus*, *Thermoactinomyces*, *Sebaldella*, and *Pantoea* were no longer dominant, with *Streptomyces* emerging as the dominant genus. In H26-ZF, *Bacillus*, *Sebaldella*, and *Pantoea* were no longer dominant, while *Weissella* became dominant. In H30-ZF, only *Sebaldella* and *Pantoea* were no longer dominant. The most significant differences among the three HTD samples were the RA of *Kroppenstedtia*, *Virgibacillus*, and *Saccharopolysopora*.

The structural differences in the dominant fungal communities of *Monascus* in fortified *Muqu* with three isolates were significantly altered. *Aspergillus* emerged as a dominant genus in these *Muqu*, with RA of 68.02% in H26-M and 34.57% in H30-M, while it constituted only 3.23% in H14-M. Additionally, other prominent genera included *Thermoascus* (32.54%), *Thermomyces* (32.50%), and *Rasamsonia* (12.59%). Dominant bacterial genera still included *Botryotrichum*, *Byssochlamys*, and *Humicola* in H26-M and H30-M, though significant differences in RA were observed (Figure 3).

In the corresponding HTD samples, the dominant fungal community underwent significant changes. In H14-ZF, RA of *Aspergillus* decreased to 29.16%, while *Thermoascus* and *Byssochlamys* reached 26.39% and 41.82%, respectively, with *Byssochlamys* becoming the most dominant fungus. In H26-ZF and H30-ZF, the RA of *Aspergillus* dropped further to 2.47% and 7.22%, respectively. Meanwhile, *Thermomyces* and *Thermoascus* became more dominant, with RAs of 64.82% and 28.52% in H26-ZF, as well as 52.52% and 29.59% in H30-ZF, respectively. Additionally, the number of dominant fungal species decreased to four.

**Figure 4:**
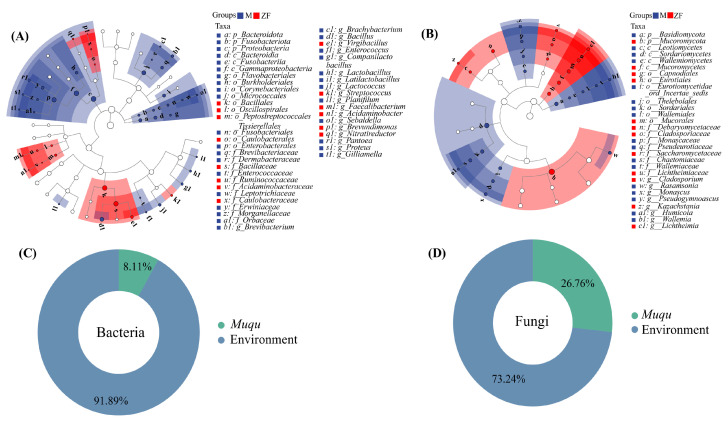
LEfSe of (**A**) bacterial and (**B**) fungal communities in *Daqu* (M and ZF represented *Muqu* and HTD); the source proportion of (**C**) bacteria and (**D**) fungi in HTD derived from the *Muqu* and the environment.

LEfSe analysis (LDA score > 2 and *p* < 0.05) revealed distinct differences in microbial communities between *Muqu* and HTD (Figure 4). A total of 19 differential bacterial genera and 8 differential fungal genera were identified across the six types of *Daqu*. HTD samples contained 6 differential bacterial genera and 3 differential fungal genera, while *Muqu* samples had 13 differential bacterial genera and 5 differential fungal genera. The predominant genera observed in *Muqu* included *Brevibacterium*, *Brachybacterium*, *Bacillus*, *Lactobacillus*, *Latilactobacillus*, *Rasamsonia*, *Monascus,* and others. In contrast, the dominant genera in HTD were *Virgibacillus*, *Streptococcus*, *Cladosporium*, *Kazachstania*, *Lichtheimia*, and others.

The results from the Source Tracker analysis, which delineated microbial origins in HTD (Figure 4C,D), indicated that 8.11% of the bacteria and 26.76% of the fungi originated from *Muqu*. The remaining microorganisms likely originated from the environment and production facilities, and warranted further investigation.

### 3.5. Correlation between Physicochemical Properties and Dominant Bacteria

**Figure 5:**
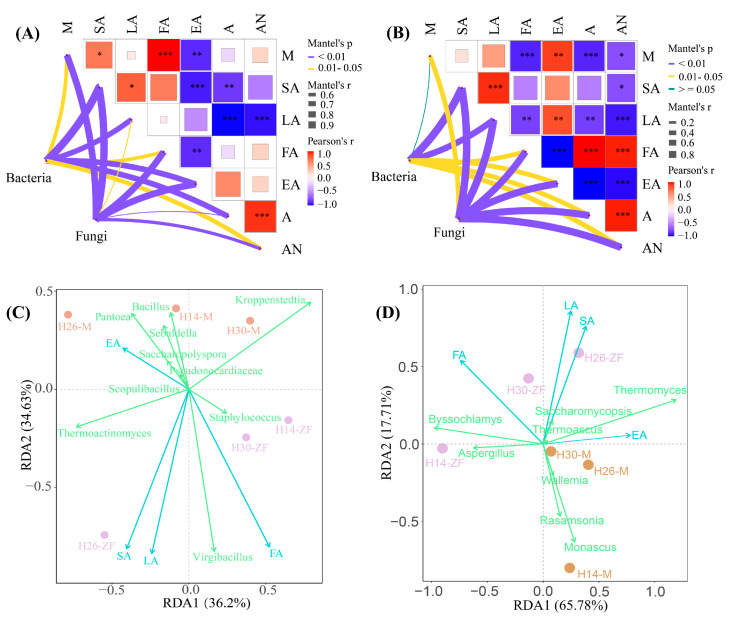
Mantel analysis between microbial communities and physicochemical indicators, (**A**) *Muqu*, (**B**) HTD; redundancy analysis between dominant genera and enzymatic properties of *Daqu*, (**C**) *Muqu*, (**D**) HTD (M: Moisture; SA: Saccharification ability; LA: Liquefaction ability; FA: Fermentation ability; EA: Esterification ability; A: Acidity; AN: Ammoniacal Nitrogen; *: *p* < 0.05; **: *p* < 0.01; ***: *p* < 0.001).

The Mantel test was employed to explore the correlation between community structure and the physicochemical properties of *Daqu* (Figure 5A,B). In *Muqu*, bacteria exhibited a significantly positive correlation with key physicochemical parameters, including saccharification ability, liquefaction ability, esterification ability, and acidity. Fungi were also significantly and positively correlated not only with saccharification ability, esterification ability, and acidity, but also with fermentation ability, moisture, and ammoniacal nitrogen. In HTD, the positive correlation between bacteria and physicochemical properties, with the exception of saccharification and liquefaction abilities, was weakened, while the positive correlation for fungi was enhanced. These findings were consistent with previous reports [11]. Redundancy analysis of the dominant genera and four types of enzyme activities (Figure 5C,D) revealed variance for bacterial and fungal communities of 70.83% and 83.49%, respectively. Most dominant bacteria, such as *Bacillus*, were positively correlated with esterification ability but negatively correlated with saccharification ability, liquefaction ability, and fermentation ability. However, *Virgibacillus*, *Thermoactinomyces,* and *Staphylococcus* showed positive correlations with these parameters. The fermentation ability of HTD was notably higher than that of *Muqu*, significantly enhancing the saccharification and liquefaction abilities of H26-ZF and H30-ZF, which may be attributed to the increased RA of *Virgibacillus* and *Staphylococcus*. Most identified fungal genera, such as *Thermomyces*, were positively correlated with esterification ability. Additionally, *Thermomyces*, *Thermoascus*, and *Saccharomycopsis* were positively correlated with saccharification and liquefaction abilities. *Byssochlamys* and *Aspergillus* were positively correlated with fermentation ability, and their increased RA in HTD contributed to a higher fermentation ability compared to *Muqu*. The correlation between these microbial genera and enzymes is multivariate and nonlinear.

The correlation analysis of physicochemical properties is illustrated in Figure 5A,B. In *Muqu*, saccharification and liquefaction abilities were significantly and positively correlated, but negatively correlated with esterification ability and acidity, which, in turn, were strongly positively correlated with ammoniacal nitrogen. In HTD, most physicochemical properties were strongly correlated with fermentation ability but negatively correlated with other physicochemical properties. The correlation between saccharification ability and liquefaction ability was consistent with that in *Muqu*, but the relationship between esterification ability and both saccharification and liquefaction ability was inversed. Due to the higher moisture in HTD, its correlation with other physicochemical properties was more pronounced.

### 3.6. Differences in Correlations between Microorganisms and Metabolites

**Figure 6:**
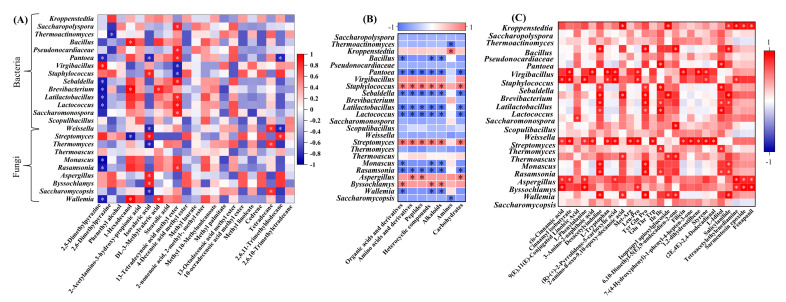
(**A**) Correlation analysis of dominant genera and characteristic volatile metabolites; (**B**) correlation analysis of dominant genera and non-volatile metabolites, (**B**) different categories and (**C**) characteristic non-metabolites (* represented *p* < 0.05).

As shown in Figure 6, the Spearman correlation coefficient was utilized to examine the relationship between microbes and their characteristic volatile metabolites. Although no reports have linked *Virgibacillus* to pyrazine synthesis, in HTD, 2,5-dimethylpyrazine exhibited a significant positive correlation with *Virgibacillus*. Phenethyl alcohol demonstrated a weak negative correlation with the most dominant bacteria. Notably, 1-Hexadecanol, known for its rose-like fragrance [25], showed a significant positive correlation with dominant bacteria, particularly *Bacillus*, *Brachybacterium*, and *Wallemia*, while it was significantly negatively correlated with *Streptomyces*. Among the four characteristic acidic volatile metabolites, isovaleric acid was negatively correlated with most dominant bacteria. Methyl 13-tetradecenoate, which imparts a honey-like fruity flavor, displayed a significant and positive correlation with most dominant bacteria, including *Saccharopolyspora*, *Pseudonocardiaceae*, *Pantoea*, and *Rasamsonia*, but was significantly negatively correlated with *Virgibacillus* and *Staphylococcus*. Among the four types of hydrocarbon volatile metabolites, only tetradecane and 2,6,11-trimethyldodecane showed significant correlations with certain dominant microbes. Hydrocarbon compounds were an important source of flavor in *Baijiu* [26,27]. However, their contribution to the overall aroma profile of *Baijiu* has been rarely reported to date.

The correlation analysis between dominant genera and non-volatile metabolites is presented in Figure 6B. With the exception of amines, *Staphylococcus* and *Streptomyces* exhibited a positive correlation with all detected non-volatile components. In contrast, *Pantoea*, *Sebaldella*, *Latilactobacillus*, *Lactococcus,* and *Rasamsonia* demonstrated significant negative correlations. *Kroppenstedtia* was the only genus that showed a significant positive correlation with amines. *Aspergillus* was strongly and positively correlated with amino acids and their derivatives, peptides, and carbohydrates, while *Byssochlamys* displayed a significant positive correlation with organic acids and their derivatives, heterocyclic compounds, and alkaloids.

These dominant genera were significantly positively correlated with 26 characteristic non-volatile metabolites (Figure 6C). Among these, Desacetylvindoline, Tyr-Gln-Pro, Ile-Ile, L-Iditol, and Salicylanilide were the five compounds strongly correlated with the dominant genera. Desacetylvindoline, a type of terpenoid alkaloid, was found to be significantly more abundant in *Muqu*, potentially due to the higher RA of *Lactobacillus*, which is known for its ability to degrade alkaloids. Peptides, such as Tyr-Gln-Pro and Ile-Ile, were also significantly more abundant in *Muqu*. Peptides in HTD play a crucial role in microbial diversity and the production of key metabolites during the fermentation process in HTD [28,29]. L-Iditol, a sugar alcohol carbohydrate, served as a characteristic flavor component that differentiates various grades of HTD [30], with the highest content observed in H26-M and H30-ZF.

### 3.7. Function Prediction of Key Metabolic Pathways of Daqu

**Figure 7:**
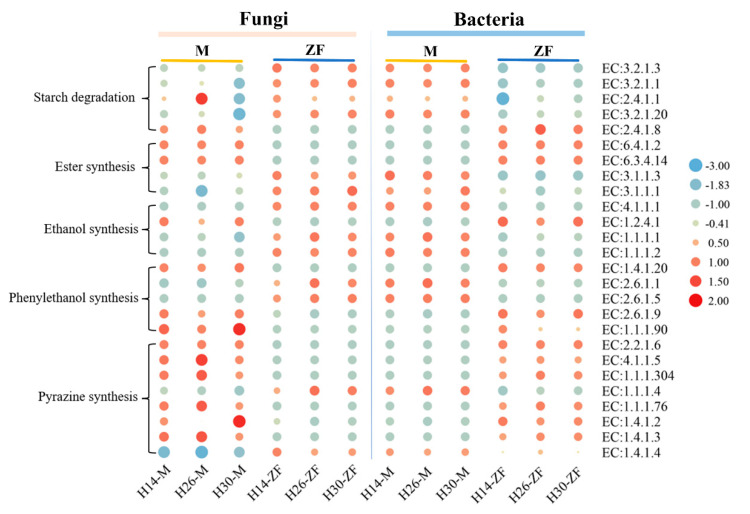
Differential analysis of key enzymes in the *Daqu*.

The PICRUSt2-predicted gene annotation results, which illustrate the differences in key enzymes of metabolic pathways between *Muqu* and HTD, are presented in Figure 7. The pathways primarily involved the degradation of starch and the synthesis of esters, ethanol, phenethyl alcohol, and pyrazine compounds. Five enzymes were annotated for starch degradation, showing that fungi in HTD contributed higher enzyme abundances compared to *Muqu*, while bacterial enzymes exhibited the opposite pattern. This suggested that the starch hydrolysis capability in HTD is mainly driven by fungi, whereas in *Muqu,* bacteria played a more significant role. Four enzymes were annotated for ester synthesis, with EC:6.4.1.2 and EC:6.3.4.14 predominantly produced by fungi in *Muqu* and bacteria in HTD, whereas EC:3.1.1.3 and EC:3.1.1.1 were mainly contributed by bacteria in *Muqu* and fungi in HTD. These findings explained the difference in ester production between *Muqu* and HTD. The synthesis of ethanol followed a similar pattern to starch hydrolysis, with fungi contributing more to ethanol synthesis in HTD, and bacteria contributing more in *Muqu*. For phenethyl alcohol synthesis, five annotated enzymes showed varied contributions: EC:1.4.1.20, EC:2.6.1.9, and EC:1.1.1.90 were more abundant in *Muqu*’s fungi than HTD, while EC:2.6.1.1 and EC:2.6.1.5 exhibited the opposite patterns. The synthesis of pyrazine compounds involved eight annotated enzymes, with fungi being the primary contributors in *Muqu* and bacteria in HTD. This indicated that fungi are primarily responsible for pyrazine synthesis in *Muqu*, while bacteria are more involved in HTD.

## 4. Conclusions

This study focused on HTD, utilizing various detection techniques to investigate the stability and differences in the properties of three types of fortified HTD used as *Muqu*. Significant differences were found between *Muqu* and HTD, with H30-M exhibiting the most stable starch decomposition ability, and H14-M showing relatively stable fermentation ability, acidity, and ammoniacal nitrogen, while H26-M contributed more stably to esterification ability. The metabolic profiles of *Muqu* and HTD also differed significantly, with *Muqu* having a more complex volatile profile and HTD containing higher levels of characteristic non-volatile components. Several important volatiles and non-volatile components in the *Muqu*, such as phenethyl alcohol, methyl palmitate, and methyl linoleate, as well as some amino acids and their derivatives and peptides, were still detected in HTD. Notable differences were also observed in the microbiota structure between *Muqu* and HTD. The number of cultivable bacteria in *Muqu* was significantly higher than that in HTD, while the proportion of cultivable mesophilic bacteria increased in HTD. However, the α-diversity of microbiota in *Muqu* was higher in *Muqu*. The composition of dominant genera in both *Muqu* and corresponding HTD samples was similar. Most dominant bacteria were significantly and positively correlated with 1-Hexadecanol and 26 characteristic non-volatile metabolic components. Starch hydrolysis and ethanol synthesis in HTD were mainly driven by fungi, whereas in *Muqu* bacteria played a more significant role. Additionally, pyrazine synthesis was mainly contributed by fungi in *Muqu* and bacteria in HTD.

## Figures and Tables

**Table 1:** Differences in the physicochemical properties of *Daqu*.

Sample	Moisture (g/100 g)	Saccharification Ability (mg/g·h)	Liquefaction Ability (g/g·h)	Fermentation Ability (g/0.5 g·72 h)	Esterification Ability (mg/50 g·7 d)	Acidity (mmol/10 g)	Ammoniacal Nitrogen (g/kg)
H14-M	9.94 ± 0.15 ^d^	81.73 ± 1.65 ^d^	0.05 ± 0.00 ^d^	0.11 ± 0.00 ^d^	603.95 ± 5.29 ^c^	1.74 ± 0.01 ^a^	4.92 ± 0.02 ^d^
H26-M	9.59 ± 0.20 ^e^	46.72 ± 0.43 ^e^	0.05 ± 0.00 ^d^	0.08 ± 0.00 ^f^	617.23 ± 1.93 ^b^	1.74 ± 0.01 ^a^	4.52 ± 0.01 ^e^
H30-M	9.81 ± 0.05 ^d^	108.93 ± 2.23 ^c^	0.07 ± 0.00 ^c^	0.10 ± 0.00 ^e^	602.64 ± 2.10 ^c^	1.62 ± 0.01 ^c^	3.81 ± 0.01 ^f^
H14-ZF	11.85 ± 0.19 ^c^	21.05 ± 0.26 ^f^	0.03 ± 0.00 ^e^	0.23 ± 0.02 ^a^	190.08 ± 2.04 ^e^	1.70 ± 0.01 ^b^	6.20 ± 0.01 ^a^
H26-ZF	13.09 ± 0.08 ^b^	439.86 ± 1.56 ^a^	0.36 ± 0.00 ^a^	0.20 ± 0.00 ^b^	529.09 ± 7.58 ^d^	1.29 ± 0.01 ^d^	5.35 ± 0.01 ^b^
H30-ZF	18.11 ± 0.09 ^a^	219.04 ± 2.21 ^b^	0.29 ± 0.00 ^b^	0.17 ± 0.00 ^c^	742.09 ± 16.45 ^a^	1.00 ± 0.01 ^e^	5.18 ± 0.02 ^c^

Note: Different letters in the same column represent significant differences (Duncan’s test, *p* < 0.05).

**Table 2:** Counts of cultivable microorganisms.

Sample	Bacteria (CFU/g)	Mesophilic Bacteria (CFU/g)	Fungi (CFU/g)
H14-M	(1.16 ± 0.01) × 10^9^ b	(1.13 ± 0.03) × 10^8^ a	(1.07 ± 0.22) × 10^4^ e
H26-M	(9.72 ± 0.21) × 10^8^ c	(3.63 ± 0.13) × 10^7^ d	(1.60 ± 0.22) × 10^4^ d
H30-M	(1.24 ± 0.02) × 10^9^ a	(3.97 ± 0.15) × 10^7^ c	(6.22 ± 0.13) × 10^3^ f
H14-ZF	(5.74 ± 0.12) × 10^7^ f	(3.35 ± 0.19) × 10^7^ d	(4.62 ± 0.45) × 10^5^ a
H26-ZF	(7.28 ± 0.20) × 10^7^ e	(4.01 ± 0.09) × 10^7^ c	(4.80 ± 0.58) × 10^4^ c
H30-ZF	(1.38 ± 0.03) × 10^8^ d	(1.08 ± 0.02) × 10^8^ b	(2.31 ± 0.13) × 10^5^ b

Note: Different letters in the same column represent significant differences (Duncan’s test, *p* < 0.05).

**Table 3:** Differences in community α-diversity.

Number of *Daqu*	Bacteria		Fungi	
	Chao1	Shannon	Chao1	Shannon
H14-M	211.96	4.56	59.02	2.71
H26-M	209.20	4.65	43.90	1.65
H30-M	159.09	4.22	63.00	2.42
H14-ZF	173.06	3.95	68.00	2.28
H26-ZF	274.24	3.85	66.05	1.60
H30-ZF	216.08	3.84	49.00	1.97

## Data Availability

The original contributions presented in the study are included in the article, further inquiries can be directed to the corresponding author.
